# Analyzing the CDR3 Repertoire with respect to TCR—Beta Chain V-D-J and V-J Rearrangements in Peripheral T Cells using HTS

**DOI:** 10.1038/srep29544

**Published:** 2016-07-12

**Authors:** Long Ma, Liwen Yang, Bin Shi, Xiaoyan He, Aihua Peng, Yuehong Li, Teng Zhang, Suhong Sun, Rui Ma, Xinsheng Yao

**Affiliations:** 1 Department of Immunology, Research Center for Medicine & Biology, Innovation & Practice Base for Graduate Students Education, Zunyi Medical University, Zunyi 563003, China; 2 Department of Laboratory Medicine, Zunyi Medical University, Zunyi 563003, China; 3 Department of Breast Surgery, The first Affiliated Hospital of Zunyi Medical University, Zunyi 563003, China

## Abstract

V-D-J rearrangement of the TCR—beta chain follows the 12/23 rule and the beyond 12/23 restriction. Currently, the proportion and characteristics of TCR—beta chain V—J rearrangement is unclear. We used high-throughput sequencing to compare and analyze TCR—beta chain V-J rearrangement and V-D-J rearrangement in the CDR3 repertoires of T cells from the PBMCs of six volunteers and six BALB/c mice. The results showed that the percentage of V-J rearrangement of the volunteers was approximately 0.7%, whereas that of the mice was 2.2%. The clonality of mice V-J rearrangement was significantly reduced compared with the V-D-J rearrangement, whereas the clonality of human V-J rearrangement was slightly reduced compared with the V-D-J rearrangement. V-J rearrangement in CDR3 involved the significant usage of N, S, F and L, whereas V-D-J rearrangement in CDR3 involved the significant usage of R and G. The levels of V deletion and J deletion in V-J rearrangement were significantly reduced compared with V-D-J rearrangement. TRBD and TRBJ usage in V-J rearrangement differed from that of V-D-J rearrangement, including dominant usage of TRBV and TRBJ and their pairing. Taken together, these results provide new ideas and technology for studies of V-D-J rearrangement and V-J rearrangement in the CDR3 repertoire.

The alpha-beta and gamma-delta TCRs of humans and mice are formed by germ line genetic rearrangement of the variable (V), diversity (D), joining (J), and constant (C) regions. At the TCR loci, the 3′ end of TRBV or TRBD in TCR beta chains is a heptamer (CACAGTG)-23 base pair (bp)-nonamer (ACAAAAACC) rearrangement of signal sequences (3′ TRBV 23 RSS and 3′ TRBD 23 RSS), whereas the 5′ end of TRBD or TRBJ is a nonamer-12bp-heptamer rearrangement of signal sequences (5′ TRBD 12 RSS and 5′ TRBJ 12 RSS)^1^. V-D-J recombination occurs only between segments with the 23 RSS terminal and segments with the 12 RSS terminal, and this restriction is called the 12/23 rule. The 12/23 rule predicts the occurrence of V-J rearrangement. However, the occurrence of V direct to J rearrangement is rare in the body, and this restriction is known as the B12/23 restriction^2^,^3^,^4^,^5^.

Previous studies have examined the 12/23 rule and the beyond 12/23 restriction (B 23/12 restriction) of TCR beta chain rearrangement. For example, in 2000, Bassing CH *et al*.^4^ used embryonic stem cells containing only the TCR beta gene locus modified by TRBD1 and TRBJ1 fragments to make a BALB/c mouse model (BALB/c mice with no TRBD2 and TRBJ2 cluster: TRBJ1 M3 allele) and found that TRBV mainly rearranged with TRBD1 but not with TRBJ1; therefore, the additional B 23/12 restriction played an important role in the regulation of V gene rearrangement. The authors also found that at the double-negative stage in the thymus, TRBV could not directly rearrange with TRBJ1. However, using 5′TRBJ1.2 12-RSS to replace TRBD1 12-RSS, TRBV directly rearranged with the modified TRBJ1.2. In 2007, using this experimental system, the authors used 3′TRBD1 RSS to modify TRBV14 RSS, indicating that TRBV14 could directly rearrange to TRBJ1^6^.

In a mechanistic study of the B 12/23 restriction, Tillman RE *et al*.^7^,^8^ confirmed that in the case of non-lymphocyte (CHO cell line) expressing recombinant activated gene protein (RAG) 1 and 2, TRBV 23-RSS exhibited more rearrangement with TRBD 12-RSS compared with TRBJ 12-RSS rearrangement (following B 12/23 restriction), and this bias may correlate with preferential combining of 5′TRBD 12-RSS and RAG. In 2003, Jung, D *et al*.^9^ confirmed that the B 12/23 restriction correlated with the compositions of rearrangements of RAG, TCR beta RSS and coding flanks. Additionally, the authors found that by changing heptamer/nonamer 12-RSS, the change in V-D-J rearrangement could be increased 50-fold, whereas the change of heptamer/nonamer sequence in natural states could only affect the change in V-D-J rearrangement by approximately 2- to 6-fold^10^.

Regarding the efficiency of the B 12/23 restriction, Jung, D CHO and 3T3 cell (plasmid transfection experiments *in vitro*) models revealed that TRBV 23-RSS mainly rearranges with TRBD 12-RSS and rarely with TRBJ 12-RSS. The rearrangement proportion of the TRBV family and TRBD-TRBJ ranged from 500:1 to 50:1; of these, the rearrangement proportion of TRBV18 and TRBD-TRBJ was the highest (up to 5:1). In addition, when TRBJ1.4 12RSS replaced TRBD1 12RSS, the rearrangement proportion of TRBV12 23RSS and TRBJ1.4 12RSS was upgraded from 500:1 to 100:1. These different rearrangement proportions observed in the experiment could therefore be used to investigate the mechanism and function of the B12/23 restriction for V-D-J rearrangement *in vivo*.

Direct rearrangement of 3′TRBD1 23RSS and 5′TRBD2 12-RSS also follows the 12/23 rule. In 1986, numerous studies found that TCR beta chain rearrangement of BALB/c mice and healthy volunteers existed as a TRBD1-TRBD2 fusion rearrangement^11^,^12^,^13^,^14^. Later, Jung D *et al*. confirmed that the B 12/23 restriction did not limit 3′ direct rearrangement of TRBD1 23RSS and 5′TRBD2 12RSS, and the frequency of TRBD1 fusion with TRBD2 was approximately the same as the frequency of rearrangement with TRBJ1.4^9^. Further studies found that if the use of 3′ TRBV14 23-RSS was replaced by TRBD 23-RSS, then TRBD 23-RSS could directly rearrange with TRBV14 and TRBJ. However, the rearrangement frequency of TRBV14 and TRBD1 12-RSS remained increased by 5- to 10-times compared with TRBV14 and TRBJ 12-RSS^15^. In 2014, Peepei Liu *et al*.^16^ used high-throughput sequencing (HTS) to detect the alpha-beta TCR complementarity determining region 3 (CDR3) repertoire in a large number of CDR3 sequences from 3 volunteers and found that approximately 2% of sequences exhibited a fusion rearrangement of TRBD genes in the TCR beta chain (or TRBD tandem rearrangement).

In the development of the T cells in the thymus, V-D-J rearrangement of the TCR beta chain follows the 12/23 rule and the B 12/23 restriction in vertebrates (Fig. 1), but TCR rearrangement by V direct to J has been reported in cell models and mouse models^4^,^15^. Currently, the frequency, characteristics, and significance of the CDR3 repertoire with respect to TCR beta chain V direct to J rearrangement are not clear. In this study, we used HTS technology to analyze the TCR beta chain CDR3 repertoire of PBMCs from healthy volunteers and BALB/c mice and compared and analyzed in detail the proportion and characteristics of the CDR3 repertoire with respect to V-J rearrangement and V-D-J rearrangement.

## Materials and Methods

### Human TCR beta chain CDR3 repertoire preparation and high-throughput technique sequencing

**1-1** Six healthy volunteers were informed of the purpose of peripheral blood collection and provided written informed consent. Peripheral blood was obtained by the Zunyi Medical College Hospital Laboratory. The age and sex of the volunteers were as follows: Cui1 (A1), male, 35 years old; Cui2 (A2), male, 35 years old; Zhao1 (B1), male, 46 years old; Zhao2 (B2), male, 46 years old; Chen1 (C1), male, 22 years old; Chen2 (C2), female, 22 years old. The names of the six volunteers have been masked. All six subjects were of Han ethnicity. All the research protocols were approved by the Ethics Committee of Zunyi Medical College, and all experiments were performed in accordance with the guidelines of the committee.

**Figure 1:**
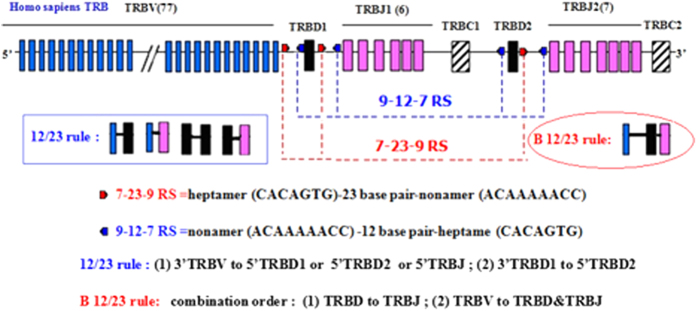
Schematic diagram of the 12/23 rule and the beyond 12/23 restriction in human TCR beta chain variable, diversity, joining (V-D-J) gene rearrangements.

**1-2** We collected 2 ml of peripheral blood from each of healthy volunteer, and PBMCs (peripheral blood mononuclear cells) were obtained using density gradient centrifugation. A QIAamp DNA MiniKit (Cat. No. 51304, QIAGEN) was used to extract genomic DNA from PBMCs, which was assessed by agarose gel electrophoresis (1%) (Sup 1 Fig. 1-A). Six genomic DNA samples were stored in a QIAsafe DNA tube (QIAGEN) and were sent to Adaptive Biotechnologies Corp (Seattle, WA, US) for sequencing. Before high-throughput sequencing, the concentration and purity of DNA of samples were confirmed for TCR CDR3 sequencing (http://www.immunoseq.com).

**1-3** A multiplex PCR system was designed to amplify rearranged TCR beta chain CDR3 from genomic DNA using 45 forward primers (TRBV) and 13 reverse primers (TRBJ) as reported in the ImmunoSEQ assay^17^. The forward and reverse primers contained the universal forward and reverse primer sequences, respectively, compatible with the GA2 cluster station solid-phase PCR at their 5′ ends. Genomic templates were amplified using an equimolar pool of the 45 TCR Vβ F primers (the “VF pool”) and an equimolar pool of the 13 TCR Jβ R primers (the “JR pool”). Data analysis for the visualization, sorting, selection, and comparison of the TCR β sequences was performed using the Illumina Genome Analyzer^16^,^18^.

### Mouse TCR beta chain CDR3 repertoire preparation and high-throughput technique sequencing

**2-1** We used six BALB/c female mice that were 8 weeks old and weighed 20 to 22 g. Mice were purchased from CAVENS Experimental Animals Ltd. The mice were tagged as M1-0, M2-0, M3-0, M1-2, M2-2 and M3-2. All the research protocols were approved by the Animal Ethics Committee of Zunyi Medical College, and all animal experiments were performed in accordance with the guidelines of the committee.

**2-2** We collected peripheral blood samples from mice using the orbital blood collection method, and PBMCs were separated by density gradient centrifugation. QIAamp DNA MiniKit (Cat. No. 51304, QIAGEN) was used to extract genomic DNA from PBMCs; genomic DNA was then identified by agarose gel electrophoresis (1%) (Sup1 Fig. 1-B). The genomic DNA samples, which were stored in QIAsafe DNA tubes (QIAGEN), were sent to Adaptive Biotechnologies Corp (Seattle, WA, US) for sequencing. Before high-throughput sequencing, the concentration and purity of the DNA of samples were confirmed for TCR CDR3 sequencing^19^.

**2-3** TCR β sequences were generated following a multiplex PCR amplification consisting of 36 forward V segments (TRBV) and 14 reverse J segment primers (TRBJ) that targeted all possible somatic combinations of the rearranged TCR beta chain CDR3. The forward and reverse primers contained the universal forward and reverse primer sequences, respectively, compatible with the GA2 cluster station solid-phase PCR at their 5′ ends. The Illumina GA2 System generated reads of 54 base pairs (bp) in length that covered the entire range of the CDR3 lengths. The 14 different Jβ gene segments each had a unique “tag” sequence downstream of the recombination signal sequence. Sequencing primers were designed to anneal to a consensus nucleotide motif observed immediately downstream of this tag. Thus, sequences starting from the Jβ segment tag routinely captured the complete CDR3 region; then, the TCR β CDR3 PCR library was loaded on an Illumina Flow Cell for sequencing on an Illumina Genome Analyzer^20^.

### Screening, analysis, and statistics of the TCR beta chain CDR3 repertoire sequences

The raw sequences in the FASTA format were analyzed using the Immuno-SEQ analyzer toolset and IMGT/High V-QUEST (version 1.3.1).

**3-1** Depending on the genetic composition characteristics of the human and mouse TCR beta chains, we removed the No results and Un-known sequences as well as out of frame sequences to define in frame (productive) sequences of every sample to perform further analysis.

**3-2** The characteristics of the TCR beta chain CDR3 repertoire sequences were defined using the Immuno-SEQ analyzer toolset and IMGT/High V-QUEST. The main characteristics were as follows: CDR3 nucleotide, CDR3 amino acid; count (reads); frequency count (%); CDR3 length; V gene name; D gene name; J gene name; V deletion; n1 insertion; D 5′ deletion; D 3′ deletion; n2 insertion; J deletion; V index; n1 index; D index; n2 index; J index; sequence status (Has stop/in frame/out frame). Additionally, the V-D-J rearrangement (clone Resolved) CDR3 repertoire was defined by “D Gene Name/D Gene Name Ties” with “TRBD1; TRBD2; TRBD1orTRBD2; TRBD1&TRBD2”, whereas the V-J rearrangement (clone Resolved) CDR3 repertoire was defined by “D Gene Name/D Gene Name Ties” with “Undefined/Unresolved TRBD”.

**3-3** We calculated the V-J and V-D-J rearrangement of the CDR3 repertoire (in frame sequences), the proportion and frequency of unique CDR3 sequences, CDR3 repertoire clonality, CDR3 AA length, CDR3 AA usage, V deletion and J deletion, and dominant TRBV-TRBJ gene pairing in the six healthy volunteers and the six BALB/c mice (Refer to the Supplemental Fig. for the statistical data of separate samples).

**3-4** Statistical Methods: TRBV gene and TRBJ gene usage as well as CDR3 AA usage were compared using χ2 test. CDR3 repertoire clonality was compared with a non-parametric one-way ANOVA and Bonferroni post-test. V and J deletions of nucleotides were compared using ANOVA. *p* < 0.05 was considered statistically significant. All statistically significant differences are indicated as * = *p* < 0.05; ** = *p* < 0.01, and *** = *p* < 0.001^18^,^21^,^22^.

## Results

### 1. Proportion, frequency, and clonality of TCR beta chain V-J and V-D-J rearrangements in the CDR3 repertoire

**Figure 2:**
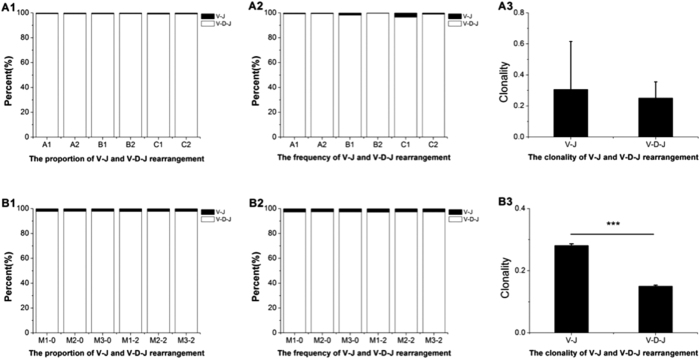
CDR3 repertoire proportion, frequency and clonality of TCR beta chain V-J and V-D-J rearrangement in six healthy volunteers (**A1, A2, A3**) and six BALB/c mice (**B1, B2, B3**). Note: The p-values of clonality were determined using a non-parametric one-way ANOVA and Bonferroni post-test. *** = *p* < 0.001.

**Figure 3:**
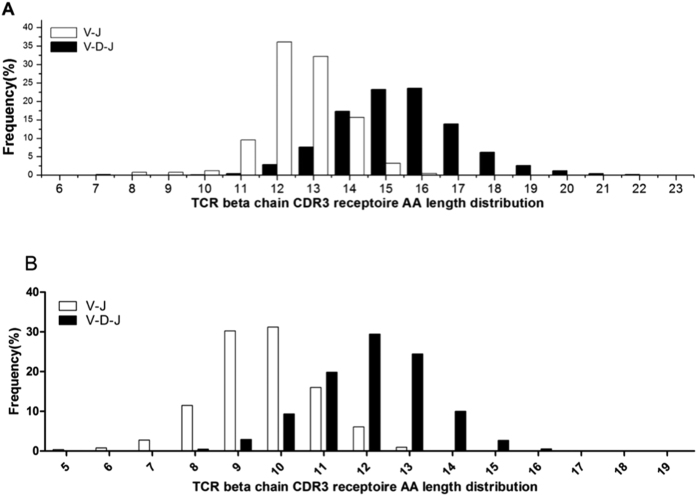
CDR3 repertoire AA length distribution of TCR beta chain V-J and V-D-J rearrangement in six healthy volunteers (**A**) and six BALB/c mice (**B**).

We calculated total and unique sequences of the TCR beta chain CDR3 repertoire in six healthy volunteers and six BALB/c mice. Next, we further calculated the unique and total sequence amounts, the proportion of unique sequences and the clonality of V-J and V-D-J rearrangement in frame sequences (Tables 1 and 2; Fig. 2). The proportion of unique V-J rearrangement sequences in the CDR3 repertoire out of the total productive unique sequences was approximately 0.7% in the volunteers (Fig. 2-A1). In contrast, the percentage was approximately 2.2% in the BALB/c mice (Fig. 2-B1). The frequency of unique V-J rearrangements in the CDR3 repertoire between different individuals was surprisingly consistent. In addition, unique sequences constituted approximately 2.9% and 5.3% of total sequences in the human V-J and V-D-J rearrangement CDR3 repertoire, respectively (Fig. 2-A2). The respective values were 8.0% and 9.7% in mice (Fig. 2-B2). In assessing the comparative analysis of the CDR3 repertoire clonality, we found that the clonality of mouse V-J rearrangement in the CDR3 repertoire was significantly reduced compared with the V-D-J rearrangement in the CDR3 repertoire (Fig. 2-B3), but the clonality exhibited increased individual differences in the six volunteers (Fig. 2A-3).

### 2. AA length distribution of the TCR beta chain V-J and V-D-J rearrangements in the CDR3 repertoire

A Gaussian CDR3 length distribution pattern was observed for both healthy volunteers and BALB/c mice. The AA length of TCR beta chain V-J rearrangements in the CDR3 repertoire was between 6 and23 aa, and the highest peak was 12 aa. Similarly, the dominant CDR3 length was 16 aa in the V-D-J CDR3 repertoire (Fig. 3-A, Sup Fig. 3). Furthermore, we also analyzed CDR3 length distribution patterns with regard to TCR beta chain V-J rearrangement in the CDR3 repertoire of BALB/c mice. The length ranged from 5 to 19 aa, and 9 aa was the highest. Similarly, the dominant CDR3 length was 12 aa with regard to V-D-J rearrangement in the CDR3 repertoire (Fig. 3-B, Sup Fig. 1).

### 3. AA usage of TCR beta chain V-J and V-D-J rearrangements in the CDR3 repertoire

**Figure 4:**
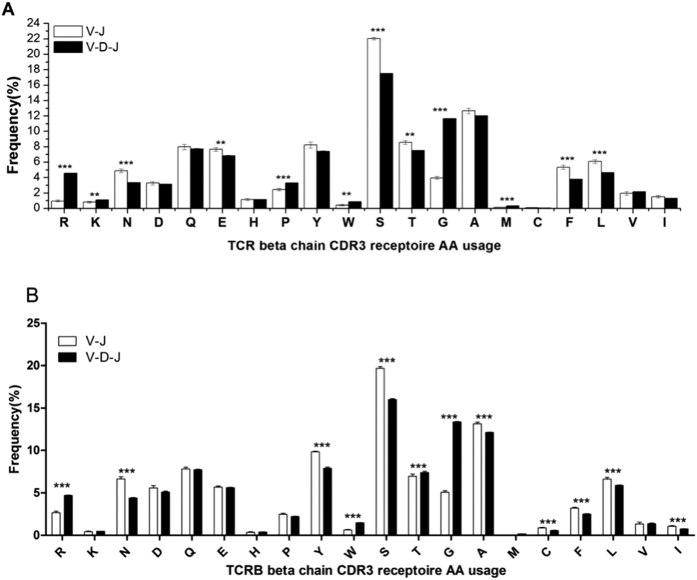
CDR3 repertoire aa usage of TCR beta chain V-J and V-D-J rearrangement in six healthy volunteers (**A**) and six BALB/c mice (**B**). Note: The p-values were determined using χ2 test. * = *p* < 0.05, ** = *p* < 0.01, *** = *p* < 0.001.

N, S, F, and L amino acids were used preferentially (*p* < 0.001) in both healthy volunteers and BALB/c mice in V-J rearrangements in the CDR3 repertoire. In contrast, R and G were the dominant amino acids in healthy volunteers, and R, G, and T were dominant in V-D-J rearrangements in the CDR3 repertoire of BALB/c mice (*p* < 0.001) (Fig. 4) (note: CDR3 repertoire AA usage does not include the 104-C and 118-F).

### 4. V and J deletions of TCR beta chain V-J and V-D-J rearrangements in the CDR3 repertoire

**Figure 5:**
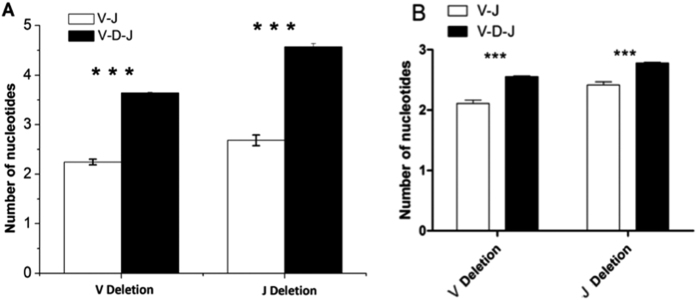
CDR3 repertoire V deletion & J deletion of the TCR beta chain V-J and V-D-J rearrangement in six healthy volunteers (**A**) and six BALB/c mice (**B**). Note: The p-values were determined using ANOVA. *** = *p* < 0.001.

V and J deletions were significantly reduced compared with V-D-J rearrangements in healthy volunteers and BALB/c mice (*p* < 0.001) (Fig. 5, Sup Fig. 2, Sup Fig. 4).

### 5. TRBV, TRBJ usage, and TRBV-TRBJ gene pairing of the TCR beta chain V-J and V-D-J rearrangements in the CDR3 repertoire

**Figure 6:**
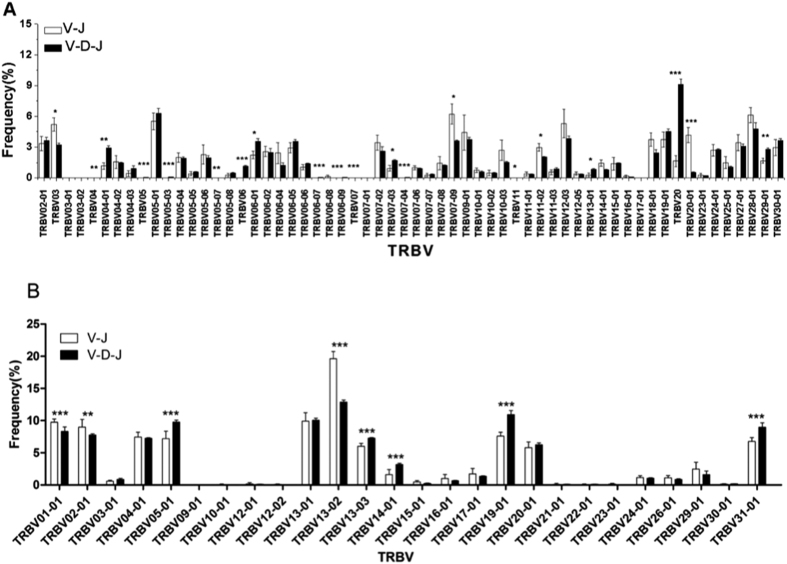
CDR3 repertoire with TRBV usage of TCR beta chain V-J and V-D-J rearrangement in six healthy volunteers (**A**) and six BALB/c mice (**B**). Note: The p-values were determined using χ2 test. * = *p* < 0.05, ** = *p* <0.01, *** = *p* <0.001.

**Figure 7:**
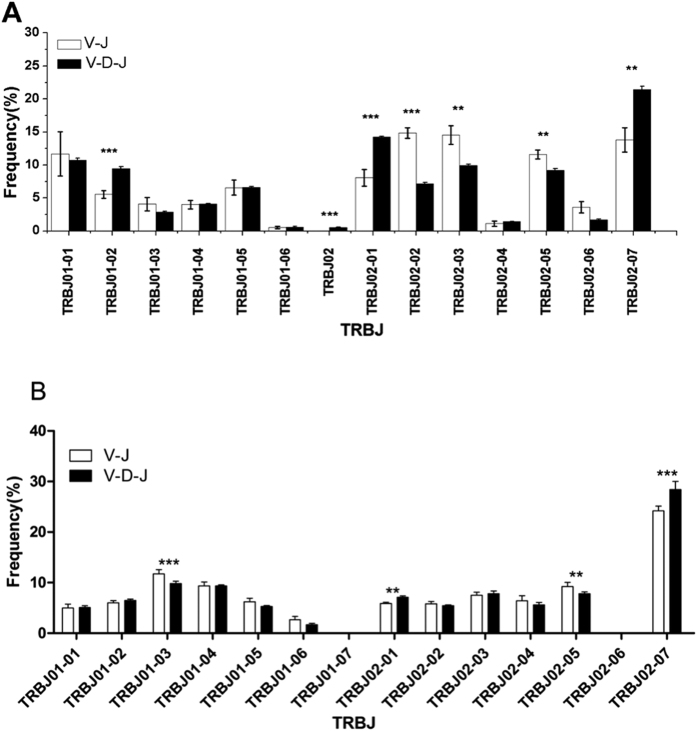
CDR3 repertoire with TRBJ usage of TCR beta chain V-J and V-D-J rearrangement in six healthy volunteers (**A**) and six BALB/c mice (**B**). Note: The p-values were determined using χ2 test. * = p < 0.05, ** = *p* < 0.01, *** = *p* < 0.001.

**Figure 8:**
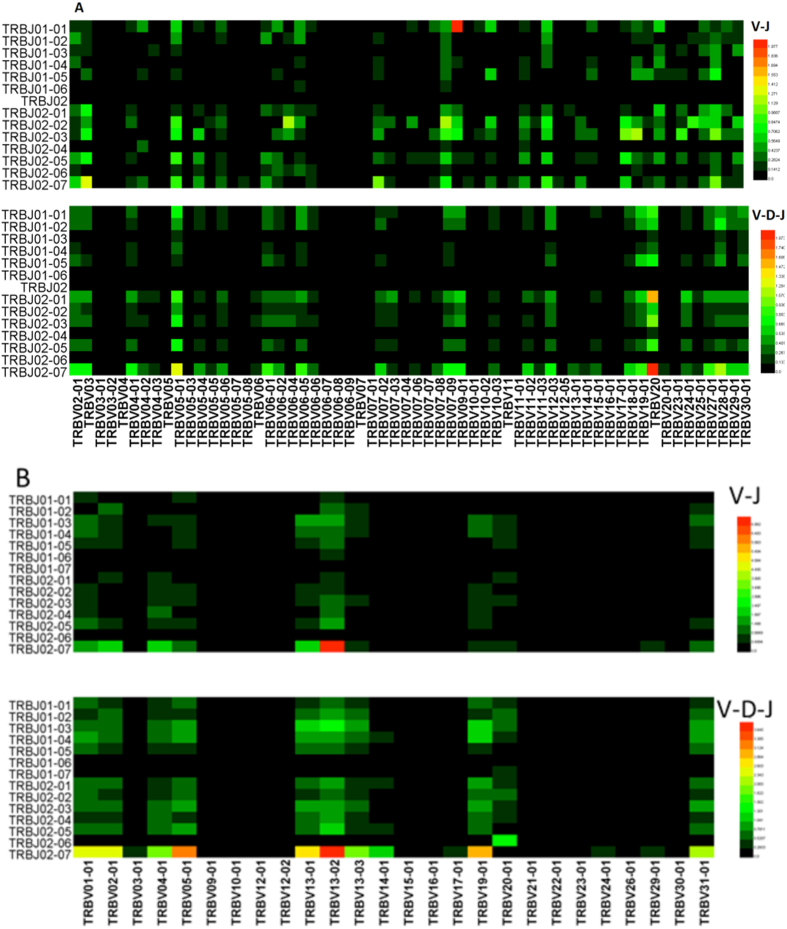
CDR3 repertoire with TRBV and TRBJ pairing of TCR beta chain V-J and V-D-J rearrangement in six healthy volunteers (**A**) and six BALB/c mice (**B**).

**5-1** In healthy volunteers, the preferential use of TRBV in the TCR beta chain V-J rearrangement CDR3 repertoire involved TRBV20-01 (Fig. 6-A) and TRBJ02-02 (Fig. 7-A), which were significantly increased compared with V-D-J rearrangement. The top 3 TRBV-TRBJ preferential gene pairings were TRBV09-01/TRBJ01-01; TRBV03/TRBJ02-07; and TRBV07-09/TRBJ02-02 (Fig. 8-A). However, in the healthy volunteers, preferential use of TRBV05, TRBV05-03, TRBV06, TRBV06-07, TRBV06-09, TRBV07-04, TRBV20 (Fig. 6-A), TRBJ01-02, TRBJ02, and TRBJ02-01 (Fig. 7-A) was notably increased in TCR beta chain V-D-J rearrangement compared with V-J rearrangement in the CDR3 repertoire (*p* < 0.001). The top 3 TRBV-TRBJ preferential gene pairings were TRBV20/BJ02-07; TRBV20/TRBJ02-01; and TRBV05-01/TRBJ02-07 (Fig. 8-A).

**5-2** Preferential use of TRBV in the BALB/c mice TCR beta chain V-J rearrangement observed in the CDR3 repertoire involved TRBV01-01, TRBV13-02 (Fig. 6-B) and TRBJ01-03 (Fig. 7-B), which were significantly increased compared with V-D-J rearrangement. The top 3 TRBV-TRBJ preferential gene pairings were TRBV13-02/TRBJ02-07; TRBV04-01/TRBJ02-07; and TRBV13-01/TRBJ02-07 (Fig. 8-B). In contrast, the high-frequency usage of TRBV in the BALB/c mice TCR beta chain V-D-J rearrangement observed in the CDR3 repertoire involved TRBV05-01, TRBV13-03, TRBV14-01, TRBV19-01, TRBV31-01 (Fig. 6-B) and TRBJ02-07 (Fig. 7-B), which were significantly increased compared with V-J rearrangement (*p* < 0.001). The top 3 TRBV-TRBJ preferential gene pairings were TRBV13-02/TRBJ02-07; TRBV05-01/TRBJ02-07; and TRBV19-01/TRBJ02-07 (Fig. 8-B).

## Discussion

The diversity of TCRs can contribute to the rearrangement of germ-line gene variable (V), diversity (D), and joining (J) gene segments. In addition, N and P addition during the V-D-J rearrangement follows the 12/23 rule and the beyond 12/23 (B 12/23) restriction. These results indicate that the rearrangement occurs only between gene segments flanked by a 23 RSS and 12 RSS (12/23 rule). In general, the D-J rearrangement occurs first, followed by V recombining with the D-J rearrangement. The V direct to J rearrangement is also permissible by the 12/23 rule, but V direct to J rearrangement TCRs are rarely noted *in vivo* (B 12/23 restriction). Several studies have demonstrated direct V-J rearrangement in cell models and mouse models^4^,^15^. However, the proportion, characteristics, and meaning of V-direct-to-J rearrangement are unclear, and the meaning of the 12/23 rule and the B 12/23 restriction in T cell development needs further clarification using new technologies and methods.

We sequenced the TCR beta chain CDR3 repertoire of peripheral T cells from six healthy volunteers and six BALB/c mice and analyzed the composition and characteristics of each CDR3 sequence using ImmuneSEQ and IMGT high-V-quest. Certain V-J rearrangement sequences were identified in these samples. In the healthy volunteers, 0.7% of the total productive unique CDR3 sequences were V-J rearrangement sequences, and the percentages of direct V-J rearrangement sequences among the six subjects were highly similar (A-1 = 0.6%, A-2 = 0.7%, B-1 = 0.7%, B-2 = 0.7%, C-1 = 0.9%, C-2 = 0.8%). In BALB/c mice, 2.2% of the total productive unique CDR3 sequences were V-J rearrangement sequences, and the percentages of V-J rearrangement sequences among the six samples were also highly similar (M1-0 = 2.2%, M2-0 = 2.2%, M3-0 = 2.1%, M1-2 = 2.3%, M2-2 = 2.2%, and M3-2 = 2.2%). This is the first study to analyze V-J rearrangement sequences in humans and mice using HTS. Previous studies found that the percent of V-J rearrangement sequences was much lower than the common V-D-J rearrangement sequences in cell models and mouse models (i.e., the proportion was 1/500 to 1/5)^4^,^9^,^15^. Here, we sequence CDR3 using HTS; the total number of sequences was 1,000,000, and the number of unique sequences was 100,000 for each sample. The total number of sequences was 15- to 20–fold larger than the number of unique sequences for humans (Subject C1 was 33-fold). The total number of sequences was 10–fold larger than the number of unique sequences for mice (M2-0 was 17-fold). The depth of sequences for each sample was not the same, but the percentages of V-J rearrangement sequences out of the total productive unique CDR3 sequences were highly similar. The percentage of V-J rearrangement sequences in mice was 3-fold larger than in humans, which suggests that the proportion of V-J rearrangement sequences may be associated with the composition of TCR germ line gene segments and the mechanism of rearrangement in different species.

We also analyzed the proportion of unique sequences in total sequences. In six healthy volunteers, the frequency of unique sequences in total V-J rearrangement sequences (2.9%) was lower than common V-D-J rearrangement sequences (5.3%), but the frequency of unique sequences in different rearrangement types was not the same. The frequency of unique sequences in total V- J rearrangement sequences was higher than common V-D-J rearrangement sequences in Subjects A2 and B2 (Table 1 and Fig. 2-A2). In the six BALB/c mice, the frequency of unique sequences in total direct V-J rearrangement sequences (8.0%) was also lower than common V-D-J rearrangement sequences (9.7%), and the frequency of unique sequences in different rearrangement types was similar (Table 2 and Fig. 2-B2). We also compared the clonality of different samples and found that the clonality of the V-J CDR3 repertoire was considerably reduced compared with the V-D-J CDR3 repertoire in mice (Fig. 2-B3). The clonality of the V-J CDR3 repertoire was reduced compared with the V-D-J CDR3 repertoire in the six volunteers, but the clonality of the six volunteers exhibited differences (Fig. 2A-3). The different clonalities of the V-J CDR3 repertoire and the V-D-J CDR3 repertoire suggested that the D segment was responsible for generating the high CDR3 repertoire. The difference in clonality among the six volunteers, but not the six BALB/c mice, suggested that the direct V-J rearrangement sequences may be associated with special immune responses.

From the analysis of the length of the CDR3 repertoire in the six healthy volunteers, we found that the length of the V-J CDR3 repertoire exhibited a Gaussian distribution with a peak of 12 aa in a range of 6 to 23 aa, and the length of the V-D-J CDR3 repertoire exhibited a Gaussian distribution with a peak of 16 aa in a range of 6 to 23 aa. In the six BALB/c mice, the length of the V-J CDR3 repertoire also exhibited a Gaussian distribution with a peak of 9 aa in a range of 5 to 19 aa, and the length of the V-D-J CDR3 repertoire showed a Gaussian distribution with a peak of 12 aa in a range of 6 to 23 aa. These results suggest that the length of the V-D-J CDR3 repertoire was longer than the V-J CDR3 repertoire, and this phenomenon may be associated with the D segments and the differences in N/P addition (Fig. 5).

From the analysis of the aa usage of the CDR3 repertoire (not including 104–C and 118-F), we found that N, S, F, and L were frequently used aa in the V-J CDR3 repertoire of the healthy volunteers and BALB/c mice. In addition, frequently used aa in the V-D-J CDR3 repertoire included R and G in the healthy volunteers and R, G, and T in the BALB/c mice (Fig. 5). The aa sequences of TRBD were GTGG for human TRBD1 (EMBL and IMGT; accession number K02545), GTSGR for human TRBD2 (EMBL and IMGT; accession number M14159), and GTGG for mouse TRBD1 and TRBD2 (EMBL and IMGT; accession numbers X00933 and X00934). The likely main source of the frequent usage of R, G, and T was TRBD. The frequent use of aa in the V-J CDR3 repertoire of the healthy volunteers and BALB/c mice was highly similar (N, S, F, and L), suggesting that the N/P addition of the V-J CDR3 repertoire may be similar in humans and mice. In the healthy volunteers and mice, we also found that both the V deletion and the J deletion in the V-J CDR3 repertoire were reduced compared with the V-D-J CDR3 repertoire (Fig. 5). During T cell development, the difference between V and J deletion in the V-J CDR3 and V-D-J CDR3 repertoires remains unclear. In addition, the mechanism underlying the frequently used aa in the V-J CDR3 repertoire is also unclear. However, the phenomenon detected by our experiment could provide a basis for analyzing the mechanism of rearrangement and function of the V-J CDR3 repertoire and the TCR alpha chain.

Many research studies on the 12/23 rule and the B 12/23 restriction have suggested that the regulation of RSS, the expression of RAG and other proteins, and the TRBV/TRBJ site are also governed by these rules. We further analyzed the usage of TRBV and TRBJ and found that the usage of TRBV20-01 and TRBJ02-02 was increased in the V-J CDR3 repertoire (*p* < 0.001) of the healthy volunteers. In addition, several TRBV and TRBJ families of high usage were noted in the V-D-J CDR3 repertoire (*p* < 0.001). A similar phenomenon was observed in the BALB/c mice, whereas only the usage of TRBV01-01 and TRBV13-02 was increased in the V-J CDR3 repertoire (*p* < 0.001). In addition, several TRBV and TRBJ families exhibited frequent use in the V-D-J CDR3 repertoire (*p* < 0.001). We compared the related TRBV gene between humans and mice according to IMGT^23^; TRBV20-1 in humans and TRBV01-01 and TRBV13-02 in mice were not related. Thus, VJ rearrangement may associate with RSSs but not the TRBV sequences, and the specific mechanism needs to be further explored. In 2003, Jung D^9^ reported that the proportion between the V-D-J rearrangement and the V-J rearrangement was different in cell models, and the percent of TRBV18-D was 5-fold greater than TRBV18-J. In our experiment, TRBV20-1 in humans and TRBV01-01 and TRBV13-02 in mice were likely to occur via direct V-J rearrangement.

We analyzed the V and J pairs in the V-J CDR3 and V-D-J CDR3 repertoires. The advantage of using V-J pairs between the two repertoires was inconsistent, and the missing VJ pairs in the V-D-J CDR3 repertoire existed in the V-J CDR3 repertoire (Fig. 8). These data suggested that the mechanism of V-J rearrangement differed from V-D-J rearrangement. We found that the mechanism of V-D-J arrangement and the functions of CDR3 were influenced by the 12/23 rule and the B 12/23 restriction through the following features: the advantageous usage of TRBV/TRBJ, the advantageous VJ pairs and missing VJ pairs, the gene site of TRBV/TRBJ, and the RSS sequences.

In summary, we analyzed the TCR beta chain CDR3 repertoire of peripheral T cells from six healthy volunteers and six BALB/c mice and obtained massive CDR3 sequences using HTS. We then analyzed the CDR3 repertoire characteristics of the V-J CDR3 repertoire and the V-D-J CDR3 repertoire in terms of the proportion of unique sequences in total sequences, clonality, CDR3 length, the usage of amino acids, the usage of TRBV/TRBJ, and the usage of VJ pairs. The V-J rearrangement (clone Resolved) CDR3 repertoire obtained using HTS was defined by “D Gene Name/D Gene Name Ties” with “Undefined/Unresolved TRBD”, and these V-J CDR3 repertoires were derived from V direct to J rearrangement or new deletion/insertion gene sequences with completely replaced TRBD segments in the process of V-D-J rearrangement. These findings require further validation. For example, the high frequency V-J rearrangement in the CDR3 repertoire observed using HTS is consistent with the V direct to J rearrangement. In this study, we add new content to the classic theory of the 12/23 rule and the B 12/23 restriction and suggest new technology and methods to analyze the mechanism of V direct to J rearrangement and the function of the V-J CDR3 repertoire.

## Additional Information

**How to cite this article**: Ma, L. *et al*. Analyzing the CDR3 Repertoire with respect to TCR—Beta Chain V-D-J and V-J Rearrangements in Peripheral T Cells using HTS. *Sci. Rep.* **6**, 29544; doi: 10.1038/srep29544 (2016).

## Supplementary Material

Supplementary Information

Supplementary Information

Supplementary Information

## Figures and Tables

**Table 1:** The CDR3 repertoire with total sequences, unique sequences, in frame sequences, and proportion of TCR beta chain V-J and V-D-J rearrangement in six healthy volunteers.

NO	Total	Unique	Analyze the in frame sequence						
			Unique	Unique/Total (Frequency %)		Proportion (%)		Clonality	
				V-J	V-D-J	V-J	V-D-J	V-J	V-D-J
A-1	384953	22008	17245	105/2141 (4.9)	17140/299802 (5.7)	0.6	99.4	0.105	0.081
A-2	405628	22534	17708	129/1860 (6.9)	17579/342113 (5.1)	0.7	99.3	0.114	0.357
B-1	609445	39294	31408	231/8468 (2.7)	31177/473351 (6.6)	0.7	99.3	0.645	0.243
B-2	512630	32243	25774	176/1286 (13.7)	25598/396443 (6.5)	0.7	99.3	0.055	0.349
C-1	546293	16113	12764	118/14277 (0.8)	12646/407195 (3.1)	0.9	99.1	0.755	0.281
C-2	634150	31224	24698	191/5268 (3.6)	24507/501463 (4.9)	0.8	99.2	0.163	0.194
Total	3093099	163416	129597	950/33300 (2.9)	128647/2420367 (5.3)	0.7	99.3	0.306 + 0.309	0.251 + 0.104

**Table 2:** The CDR3 repertoire with total sequences, unique sequences, in frame sequences, and proportion of TCR beta chain V-J and V-D-J rearrangement in six BALB/c mice.

Name	Total	Unique	Analyze the in frame sequence						
			U/T	Unique/Total (Frequency %)		Proportion (%)		Clonality	
				V-J	V-D-J	V-J	V-D-J	V-J	V-D-J
M1-0	812536	86312	63131	1388/16308 (8.5)	61743/579925 (10.6)	2.2	97.8	0.046	0.023
M2-0	1087050	64645	46789	1004/20169 (5)	45785/767141 (6.0)	2.2	97.8	0.048	0.026
M3-0	468809	54299	39573	837/9052 (9.2)	38732/333666 (11.6)	2.1	97.9	0.045	0.024
M1-2	719794	78437	57501	1318/14871 (8.9)	56183/513020 (11.0)	2.3	97.7	0.045	0.023
M2-2	753775	72570	52594	1175/14521 (8.1)	51415/530842 (9.7)	2.2	97.8	0.057	0.032
M3-2	652979	77789	56774	1263/12551 (10.1)	55511/463819 (12.0)	2.2	97.8	0.041	0.023
Total	4494943	434052	316362	6985/87472 (8.0)	309369/3188413 (9.7)	2.2	97.8	0.047 + 0.005	0.025 + 0.004
