# Supplementary material for: Analyzing the CDR3 Repertoire with respect to TCR—Beta Chain V-D-J and V-J Rearrangements in Peripheral T Cells using HTS
Source: Sci Rep. 2016 Jul 12;6:29544. doi: 10.1038/srep29544 (PMC4941575; doi:10.1038/srep29544)
Supplement: Supplementary Information [file srep29544-s1.pdf]

Analyzing the CDR3 Repertoire with respect to TCR—Beta Chain V-D-J and V-J Rearrangements in Peripheral T Cells using HTS

Long Ma[1®], Liwen Yang[1®], Bin Shi[2®], Xiaoyan He[1], Aihua Peng[1], Yuehong Li[1], Teng Zhang[1], Suhong Sun[3], Rui Ma[1], Xinsheng Yao[1]*

1. Department of Immunology, Research Center for Medicine & Biology, Innovation & Practice Base for Graduate Students Education, Zunyi Medical University, Zunyi 563003, China,

2. Department of Laboratory Medicine, Zunyi Medical University, Zunyi 563003, China,

3. Department of Breast Surgery, The first Affiliated Hospital of Zunyi Medical University, Zunyi 563003, China

*immunology01@126.com

® These authors contributed equally to this work.

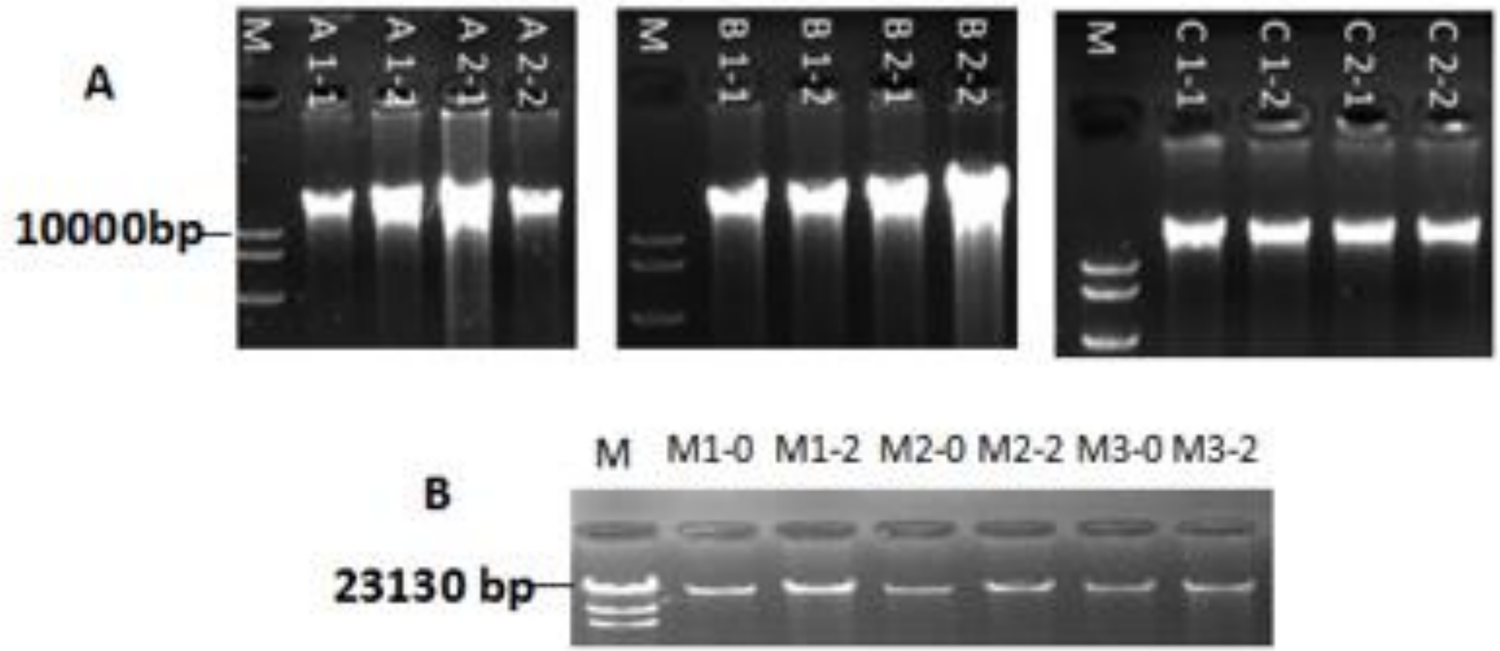

Figure 1. The 1% agarose gel electrophoresis diagram of genomic DNA from peripheral blood mononuclear cells (PBMCs) in the six healthy volunteers **(A)** and six BALB/c mice **(B).** Note: M, marker; notation of six healthy volunteers: A-1, A-2, B-1, B-2, C-1, C-2; notation of six BALB/c mice: M1-0, M1-2, M2-0, M2-2, M3-0, M3-2.

Table 1 Correspondence TRBV families between human and mcie.

<table>
<tr><th>Human TRBV</th><th>Mice TRBV</th><th>Human TRBV</th><th>Mice TRBV</th><th>Human TRBV</th><th>Mice TRBV</th></tr>
<tr><td>TRBV2-01</td><td>TRBV3-01</td><td>TRBV05</td><td rowspan="10">TRBV12-02</td><td>TRBV07-08</td><td rowspan="6">TRBV14-01</td></tr>
<tr><td>TRBV03</td><td rowspan="3">TRBV4-01</td><td>TRBV05-01</td><td>TRBV11</td></tr>
<tr><td>TRBV03-01</td><td>TRBV05-03</td><td>TRBV11-01</td></tr>
<tr><td>TRBV03-02</td><td>TRBV05-04</td><td>TRBV11-02</td></tr>
<tr><td>TRBV04</td><td rowspan="4">TRBV5-01</td><td>TRBV05-05</td><td>TRBV11-03</td></tr>
<tr><td>TRBV04-01</td><td>TRBV05-06</td><td>TRBV14-01</td></tr>
<tr><td>TRBV04-02</td><td>TRBV05-07</td><td>TRBV07</td><td rowspan="12">TRBV16-01</td></tr>
<tr><td>TRBV04-03</td><td>TRBV05-08</td><td>TRBV07-01</td></tr>
<tr><td>TRBV06</td><td rowspan="7">TRBV8-01</td><td>TRBV09-01</td><td>TRBV07-02</td></tr>
<tr><td>TRBV06-01</td><td>TRBV13-01</td><td>TRBV07-03</td></tr>
<tr><td>TRBV06-04</td><td>TRBV06-02</td><td rowspan="5">TRBV13-02</td><td>TRBV07-04</td></tr>
<tr><td>TRBV06-05</td><td>TRBV10-01</td><td>TRBV07-06</td></tr>
<tr><td>TRBV06-06</td><td>TRBV10-02</td><td>TRBV07-07</td></tr>
<tr><td>TRBV06-07</td><td>TRBV10-03</td><td>TRBV07-09</td></tr>
<tr><td>TRBV06-09</td><td>TRBV25-01</td><td>TRBV12-03</td></tr>
<tr><td>TRBV27-01</td><td rowspan="2">TRBV10-01</td><td>TRBV24-01</td><td rowspan="2">TRBV29-01</td><td>TRBV12-05</td></tr>
<tr><td>TRBV06-08</td><td>TRBV28-01</td><td>TRBV17-01</td></tr>
<tr><td>TRBV16-01</td><td>TRBV21-01</td><td>TRBV20</td><td rowspan="3">TRBV30-01</td><td>TRBV18-01</td></tr>
<tr><td>TRBV23-01</td><td>TRBV26-01</td><td>TRBV20-01</td><td>TRBV15-01</td><td>TRBV17-01</td></tr>
<tr><td>TRBV30-01</td><td>TRBV31-01</td><td>TRBV29-01</td><td>TRBV19-01</td><td>TRBV19-01</td></tr>
</table>
