# Supplementary material for: Analyzing the CDR3 Repertoire with respect to TCR—Beta Chain V-D-J and V-J Rearrangements in Peripheral T Cells using HTS
Source: Sci Rep. 2016 Jul 12;6:29544. doi: 10.1038/srep29544 (PMC4941575; doi:10.1038/srep29544)
Supplement: Supplementary Information [file srep29544-s3.pdf]

Analyzing the CDR3 Repertoire with respect to TCR—Beta Chain V-D-J and V-J Rearrangements in Peripheral T Cells using HTS

Long Ma[1®], Liwen Yang[1®], Bin Shi[2®], Xiaoyan He[1], Aihua Peng[1], Yuehong Li[1], Teng Zhang[1], Suhong Sun[3], Rui Ma[1], Xinsheng Yao[1]*

1. Department of Immunology, Research Center for Medicine & Biology, Innovation & Practice Base for Graduate Students Education, Zunyi Medical University, Zunyi 563003, China,

2. Department of Laboratory Medicine, Zunyi Medical University, Zunyi 563003, China,

3. Department of Breast Surgery, The first Affiliated Hospital of Zunyi Medical University, Zunyi 563003, China

*immunology01@126.com

® These authors contributed equally to this work.

Supplement Fig of six healthy volunteers

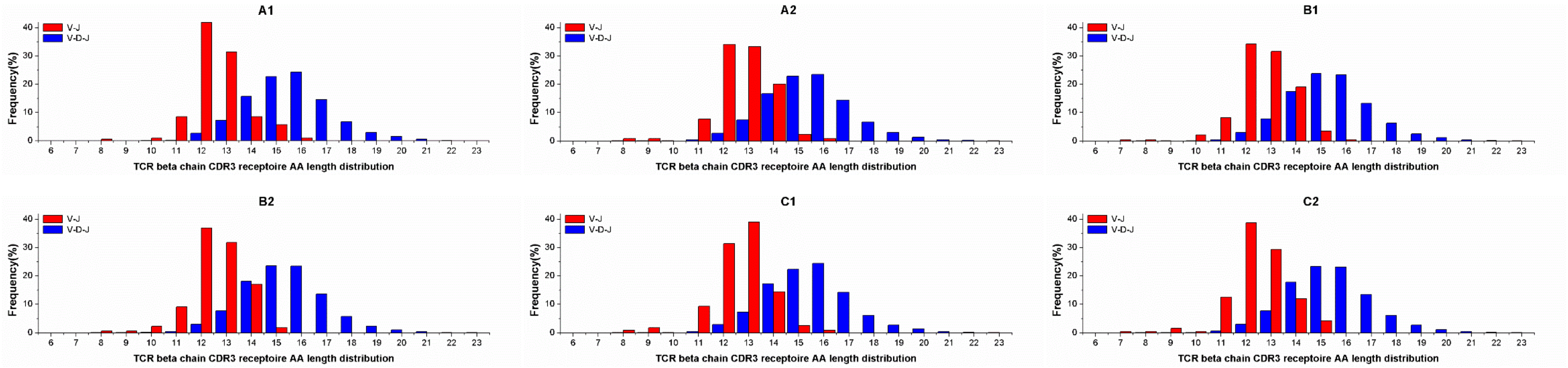

Sup **Fig 1** The CDR3 repertoire AA length distribution of TCR beta chain V-D-J and V-J rearrangement in six healthy volunteers

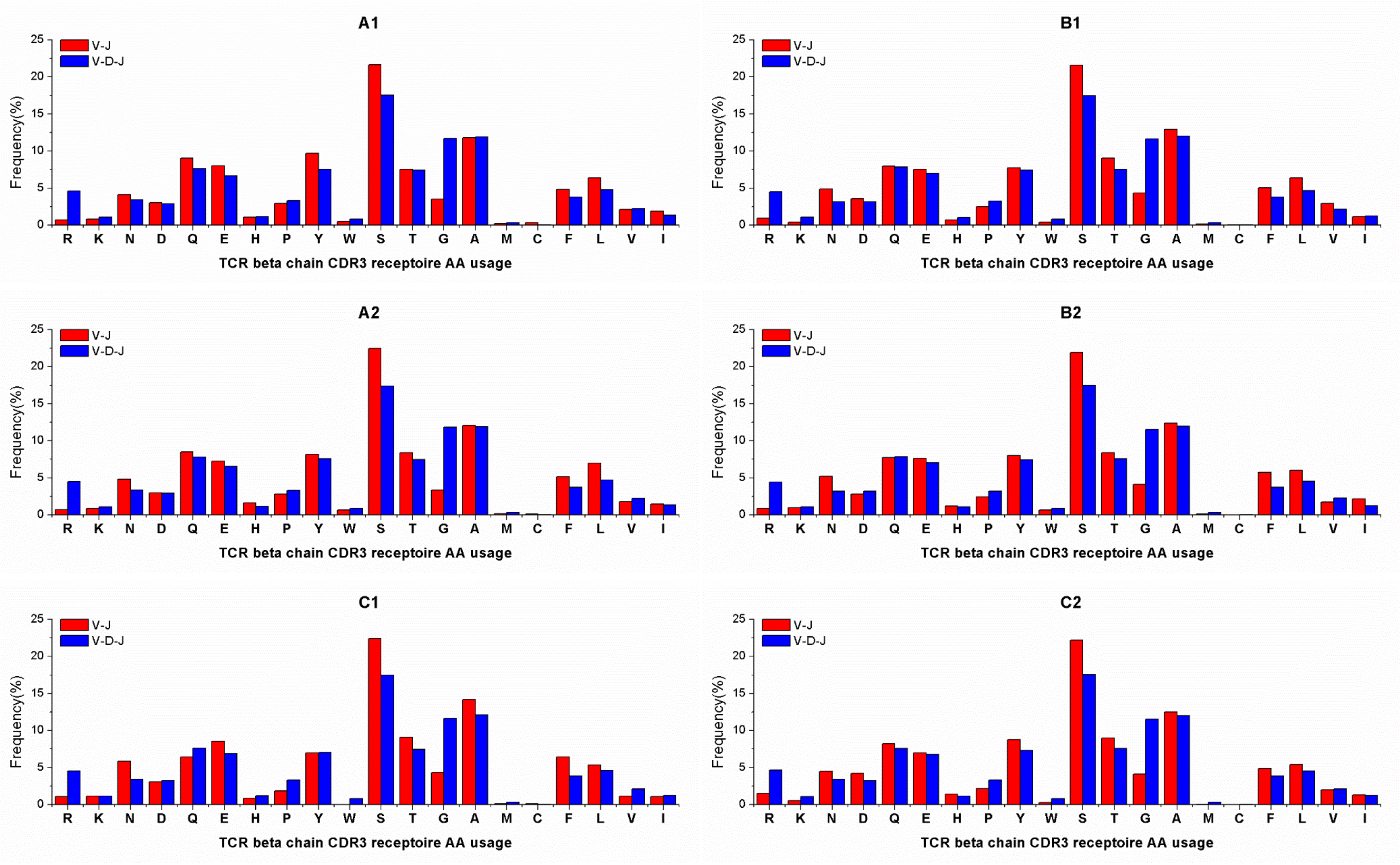

Sup **Fig 2** The CDR3 repertoire AA usage of TCR beta chain V-D-J and V-J rearrangement in six healthy volunteers

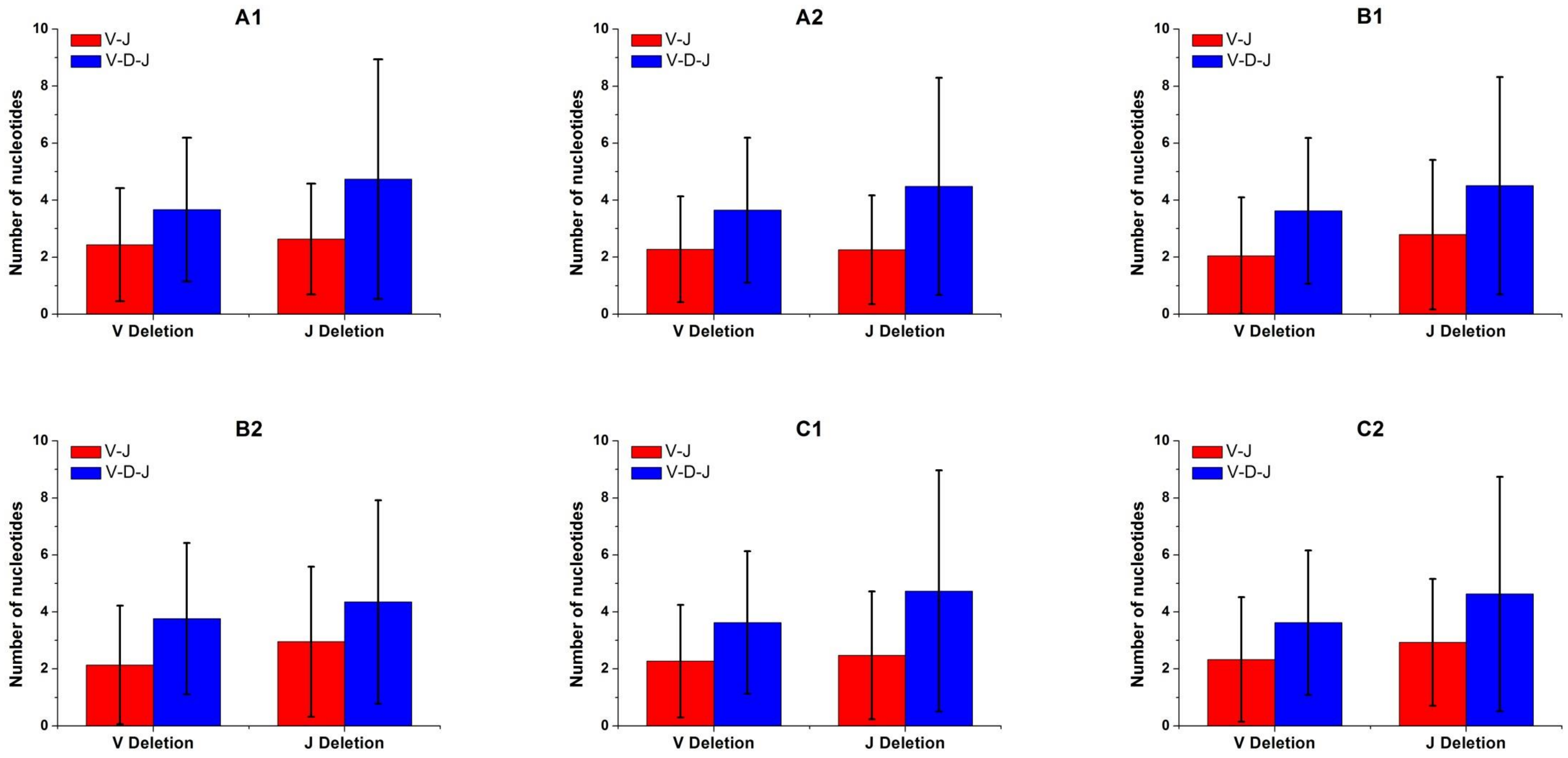

Sup **Fig 3** The V Deletion & J Deletion of TCR beta chain V-D-J and V-J rearrangement CDR3 repertoire in six healthy volunteers

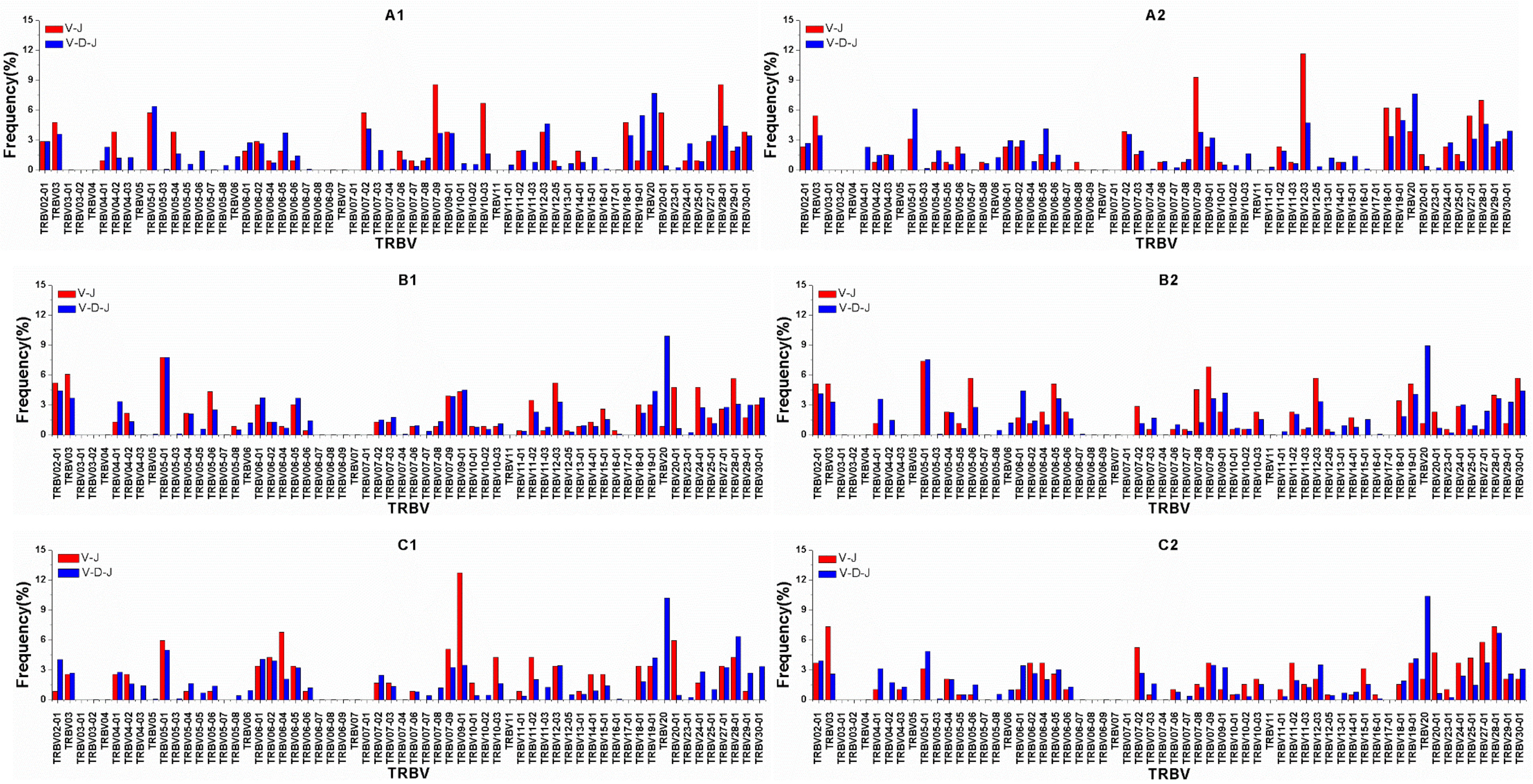

Sup **Fig 4** The CDR3 repertoire with TRBV usage of TCR beta chain V-D-J and V-J rearrangement in six healthy volunteers

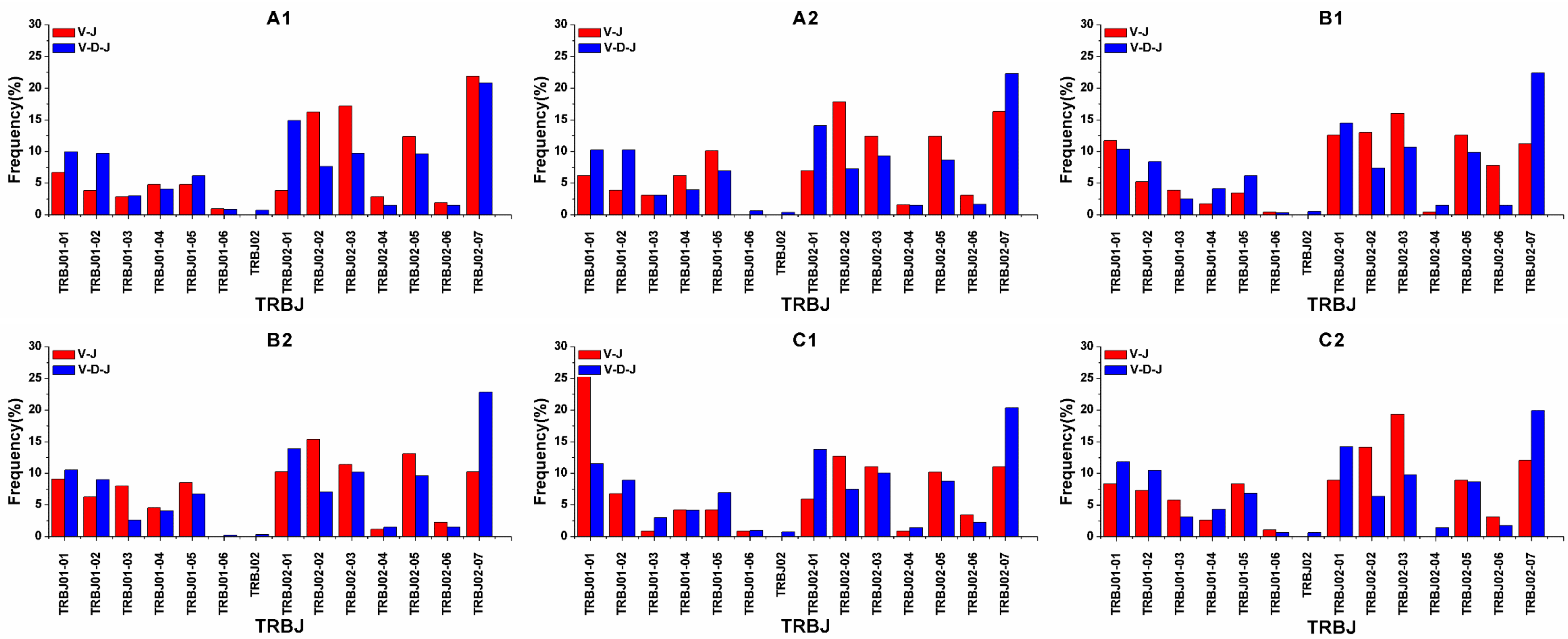

Sup **Fig 5** The CDR3 repertoire with TRBJ usage of TCR beta chain V-D-J and V-J rearrangement in six healthy volunteers

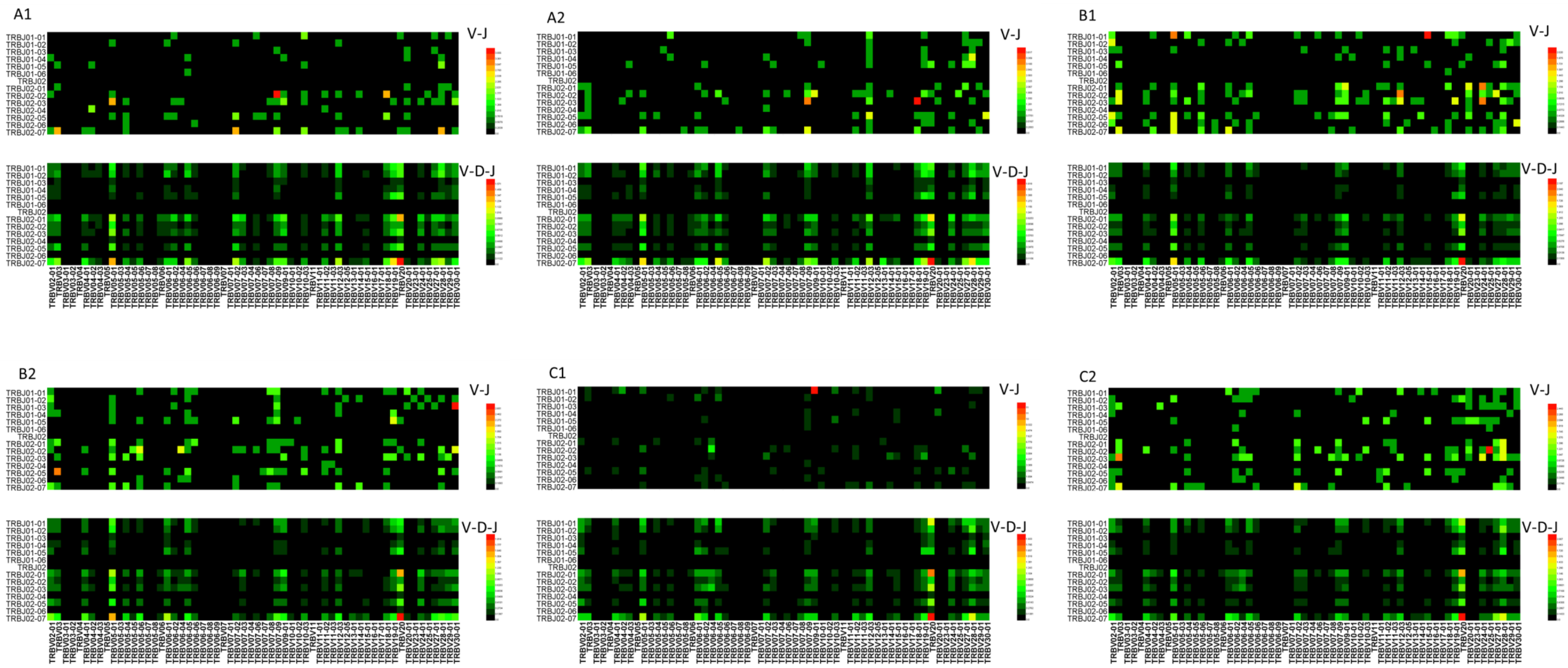

Sup **Fig 6** The CDR3 repertoire with TRBV and TRBJ pairing of TCR beta chain V-D-J and V-J rearrangement in six healthy volunteers
